# Electoral participation of people with and without disabilities in urban communities in Cameroon and Senegal

**DOI:** 10.4102/ajod.v13i0.1399

**Published:** 2024-10-16

**Authors:** Vladimir Y. Pente, Anita Jeyam, Stevens Bechange, Emma Jolley, Anne Roca, Sandra R. Dossou, Khady Ba, Joseph Oye, Salimata Bocoum, Laurene Leclercq, Elena Schmidt

**Affiliations:** 1 Sightsavers, Cameroon Country Office, Yaounde, Cameroon; 2 Sightsavers, Haywards Heath, United Kingdom; 3 Sightsavers, Senegal Country Office, Dakar, Senegal

**Keywords:** people with disability, political participation, Cameroon, Senegal, Africa

## Abstract

**Background:**

The right to participate in political processes is fundamental to democratic governance, economic development and human rights.

**Objectives:**

We assessed participation in political processes and also explored factors associated with voting at the most recent election for people with and without disabilities.

**Method:**

We conducted cross-sectional survey in four cities in Senegal and three in Cameroon in 2021. Disability was assessed using the Washington Group Short Set of questions. Univariate and multiple regression analyses were conducted to identify the factors associated with voting at the most recent elections.

**Results:**

Among 4180 participants in Cameroon and 4171 in Senegal, disability prevalence was 9.77% and 10.89%, respectively. More than half of the participants had voted at the most recent elections in both Cameroon (52.31%) and Senegal (58.27%). Participants with an interest in politics, having all the key documents or registered with a political party were more likely to have voted in both countries. Adjusting for socio-demographic characteristics, people with disabilities were less likely to have voted compared to those without disabilities in Cameroon (odds ratio [OR] = 0.58 [0.40, 0.84]) and in Senegal (OR = 0.36 [0.26, 0.44]).

**Conclusion:**

There is an urgent need to address the socio-political and environmental factors that have been identified so as to close the disability gaps in voting and ensure equitable opportunities and levels of political participation between people with and without disabilities.

**Contribution:**

This article contributes to the existing knowledge base on the political participation of people with and without disabilities in Cameroon and Senegal.

## Introduction

### Background

The right to participate in politics and public life is fundamental to achieving democratic governance, social inclusion and economic development and to realising human rights (Pente et al. 2022a; 2022b; United Nations 1967; United Nation General Assembly 1948). Political participation refers to voluntary activities or actions undertaken by ordinary people (Brady 1999:737–801; Ekman & Amna 2012:283–300) or the mass public (Uhlaner 2015: 504–508) to influence public policy, either directly or by influencing the choice of selection of people making policies.

Political participation can take on many different forms: *voting* (in a presidential, municipal, referendum, party election); *campaign activity* (including membership in or work for political parties and organisations as well as donating money to such parties or groups); *contacting public officials* and *cooperative or communal activities* (basically as all forms of engagement that focus on issues in the local community) (Ekman & Amna 2012:283–300; Verba & Nie 1987). Voting has long been perceived as the primary way for citizens to make their voice heard in the political system, and voter turnout has been described as the most commonly used measure of civic participation (Ekman & Amna 2012:283–300).

We draw on the seminal work of Van Deth (2014:349–367) and concepts from the Civic Voluntarism Model in our discussion of the factors that influence the participation of people with and without disabilities in political processes (Van Deth 2014:349–367; Verba, Schlozman & Brady 1995). The Civic Voluntarism Model is one of many frameworks within the vast landscape of civic participation theories that seeks to explain the determinants of political participation by focussing on *resources, engagement* and *recruitment* (Verba et al. 1995). Resources such as time, money, education are essential assets for engaging in political activities (Barkan 2004:913–937). Resources help you to have the money to pay the stamps for the birth certificate and identification (ID) card and also have the knowledge about the importance of those document through education. Engagement creates an essential connection to communities and their individual members through political interests, partisanship and political ideology. Recruitment encourages people to engage in political activity, whether or not directly asked (Verba et al. 1995).

### Political participation in the general population

In a democratic system, voting in national and local elections is a fundamental civic process through which the citizens of a country choose the people who represent them in policymaking and make decisions on their behalf. In order to vote in elections or referendums, eligible people need to be registered with the national electoral commission. The purpose of a registration process is to check eligibility, identify the appropriate location for the individual to vote and to reduce the potential of voting more than once (UN Women & UNDP 2015). Systems and criteria for eligibility may vary, but typically as part of this process, an individual who wishes to register must prove their identity, that they are a citizen and that they are of age to vote (Carter Centre 2013).

The way in which voters can prove their identity varies across countries. In many countries, proof of identity is assured through the possession of a birth certificate and/or a national identity card (Carter Centre 2013). In some countries, other documents are allowed to prove identity such as driving licence, army force ID card, student card, employee ID card (Highton 2017:149–167). In Cameroon and Senegal, where this study was conducted, national identity cards are used for people to both register to vote and vote (Carter Centre 2013; Direction Générale des Elections 2018; Passanti 2021:515–525).

Previous studies of political participation show that the availability of documents required to prove one’s identity can have a significant impact on the ability of citizens to register to vote and thus influence who is able to cast their vote (Okinda, Ojwang & Nyambuga 2020:63–87). Documents that are expensive or time consuming to obtain may be held by fewer citizens, disproportionately favouring those with resources or living in more accessible locations. While robust registration processes are recognised as important (Wolf et al. 2017), there are cases where the requirements may inadvertently disenfranchise certain groups of individuals who may find it harder to access the documents required for registration or to navigate the registration process. As an example, it has been reported that people with disabilities (European Union Agency for Fundamental Rights 2014; Electoral Institute for the Sustainability of Democracy in Africa 2010; International Foundation for Electoral Systems et al. 2018), women (Afrobarometer 2021:236; Electoral Institute for the Sustainability of Democracy in Africa 2010; UN Women & UNDP 2015) and the homeless (Heringa & Nguyen 2020; Tucker, De León & Mccool 2020) face difficulties in fulfilling the requirements to register to vote.

Studies from other settings have reported the influence of socio-demographic characteristics such as age (Jeroense & Spierings 2023:1–23; Norris 2002:19–34), gender (Fakih & Sleiman 2024:154–177; Goyal 2023:1–16), education, marital status, employment status, income (Alelaimat 2023:54–67) and residential location (Cho, Gimpel & Dyck 2006:156–167) on voters’ electoral participation. In studies conducted in sub-Saharan Africa, it has been shown that age is a significant positive predictor of voter turnout as older people are more likely to vote than the youth (Afrobarometer 2011:37; Jeroense & Spierings 2023:1–23; Norris 2002:19–34; Tambe & Kopacheva 2024:97–115). Education equips people with knowledge needed to understand politics and internalise political messages and has also been positively associated with more active voting behaviour in other settings (Ahearn, Brand & Zhou 2023:574–597; Hansen & Tyner 2021:711–735; Quintelier 2010:137–154). General interest and motivation to participate in politics are also a predictor of voting and other types of political participation. In a meta-analysis by Smets and Van Ham (2013), the association between political interest and electoral turnout was found to be significant in 85% of the reviewed studies (Smets & Van Ham 2013:344–359). Goldberg and Sciarini, argued that in the causal chain, political interest determines the likelihood of voting, which then determines the voter turnout (Goldberg & Sciarini 2023:141–160).

### Political participation and people with disabilities in Cameroon and Senegal

Like many other African countries, recent studies in Cameroon and Senegal highlight the challenges faced by people with disabilities, including exclusion from participation in political activities (Breffka et al. 2023:1085; Opoku, Mprah & Saka 2016:980–999; Thiendella Fall, Deslandes & Parent 2019:23–34). There is evidence to suggest that people with disabilities in both countries experience various types of barriers, including stigma and negative attitudes towards persons with disabilities (Opoku et al. 2016:980–999), social isolation (Opoku et al. 2017:67–75), lower levels of education (United Nations Education Scientific Cultural Organisation & Unesco Institute for Statistics 2018) and limited financial resources (Virendrakumar et al. 2018:509–538). In addition to limited accessibility to voter registration centres and polling stations (Virendrakumar et al. 2018:509–538), they may have difficulties accessing the required documents for voter registration (International Foundation for Electoral Systems & National Democratic Institute 2014; International Foundation for Electoral Systems et al. 2018). All these barriers can have a negative impact on the ability of people with disabilities to vote. However, empirical evidence on the determinants of political participation among people with disabilities and other disadvantaged groups in Cameroon and Senegal and sub-Sahara Africa more broadly continues to be limited. Both Cameroon and Senegal are signatories to the United Nations Convention on the Rights of Persons with Disabilities (UNCRPD) and both have passed national legislation, which guarantee political rights and the opportunity for people with and without disabilities to enjoy them on an equal basis with others (Republic of Cameroon 2023; Republic of Senegal 2010, 2013). However, in Cameroon, the Electoral Code does not make any clear provision to facilitate the participation of people with disabilities in the electoral process (Republic of Cameroon 2012), while in Senegal the *2010 Act* on absolute parity between men and women encourages the participation of women, particularly women with disabilities, in public life (Pente et al. 2022a; 2022b; Republic of Senegal 2010).

In this study, we use data from two large cross-sectional surveys to explore the factors that influence the electoral participation of people with and without disabilities in urban areas of Cameroon and Senegal. This study has three specific objectives: firstly, we assess the participation of people with and without disabilities in specific political processes, including membership in political parties, debating politics and voting in the most recent elections. Secondly, we examine the socio-demographic characteristics associated with the participation. Thirdly, we assess how access to the key documentation, such as birth certificates, national identity card and voter registration influence participation in elections, for different population sub-groups.

## Methods

### Study design and setting

This study, which was based on two cross-sectional surveys of randomly selected households, was conducted between May and October 2021. One survey was conducted in three cities of Cameroon (Maroua, Mbalmayo and Yaoundé) and the other in four cities of Senegal (Louga, Kaolack, Kaffrine and Pikine). These locations were purposefully selected as they were areas where Sightsavers had implemented activities related to enhancing the political participation of people with disabilities (Pente et al. 2022a; 2022b). Deploying a cross-sectional design allowed us to consider a number of characteristics or factors and determine which ones were associated with electoral participation at a specific point of time after the last election. At the time of the study, the most recent election had taken place in 2020 in Cameroon and in 2019 in Senegal.

### Study population and sampling

We included all adults of voting age (20 years and above in Cameroon and 18 years old and above in Senegal) at the most recent elections: the 2020 Parliamentarian and municipal election in Cameroon and the 2019 presidential election in Senegal. In both countries, sample size was calculated based on the total population of voting age in the studied areas: 3 809 643 people in Cameroon (National Institut of Statistics & Ministry of Public health 2016) and 4 007 16 people in Senegal (National Agency for Statistics and Demography 2014:36). The sample comprised 4627 individuals for Cameroon and 4365 individuals for Senegal. In both countries, the sample size was distributed according to the percentage of the population of each city in the total population of the country. In Cameroon, the sample size was distributed as follows: Mbalmayo 357, Maroua 1332 and Yaounde 2939. In Senegal, the sample size was distributed as follows: Pikine 747, Kaolack 2244, Kaffrine 395 and Louga 978. In both countries, a two-stage sampling methodology was used. The first stage involved the random selection of residential neighbourhoods from the selected cities based on probability proportional to size. At this stage, 94 residential neighbourhoods were selected in Cameroon and 85 in Senegal. At the second stage, a random walk was used to select households within each neighbourhood (Thompson 2006:11–24). In each cluster 50 individuals were randomly selected. All eligible adults present in the household at the time of the survey were invited to participate.

### Survey measures and data collection tools

Our primary outcome of interest was having voted at the most recent elections, which was binary, coded as ‘Yes’ or ‘No’. As covariates of interest, we considered resources and political engagement dimensions, as described in the Civic Voluntarism Model (Barkan 2004:913–937; Verba et al. 1995). These included socio-demographic characteristics (age, sex, residency, education, wealth), disability status and possession of the civic and voter documents, discussing politics and membership in political parties, coded as ‘Yes’ or ‘No’. Education was based on the highest level of formal schooling completed by a respondent.

Household wealth status was assessed using the Cameroon and Senegal Equity Tools, which classified the population into one of five asset-based wealth quintiles of the urban population (the first quintile ‘Q1’ being the poorest and the fifth quintile ‘Q5’ the richest). The wealth quintiles were then dichotomised for the purposes of analyses: Q1 and Q2 as the relatively poor group and Q3, Q4 and Q5 as the relatively wealthier group (Chakraborty et al. 2016:141–154). Disability status was assessed using the Washington Group Short Set of Questions on Disability (Washington Group on Disability Statistics 2022). Disability was determined by participants’ responses to six questions relating to six functional domains: seeing, hearing, walking and/or climbing, communication, self-care and remembering and/or concentrating. The responses were given on a four-point scale: no difficulty, some difficulty, a lot of difficulty or cannot do at all (Pente et al. 2022a; 2022b). Following the Washington Group recommendations, participants who responded ‘a lot of difficulty’ or ‘cannot do at all’ to at least one of the six questions were categorised as having a disability.

Data were collected using an interviewer administered questionnaire (S1 annex 1: data collection tool), which took between 20–30 min to complete per participant. The questionnaire comprised 4 sections: (1) socio-demographic characteristics, (2) the Cameroon and Senegal Equity Tools to assess the household relative wealth (Institut National de la Statistique/INS & ICF 2020), (3) the Washington Group Short Set of Questions on Disability adults (Washington Group on Disability Statistics 2022) and (4) the political participation questionnaire specific to the Cameroon and Senegal political participation context. All study questionnaires were translated into French and Fulfulde for Cameroon and French and Wolof for Senegal. The French, Fulfulde and Wolof versions were pilot tested prior to the survey in two residential areas of the city of Yaoundé in Cameroon and in another residential area in Dakar. In selecting the areas for pilot testing, attention was paid to the tool specification, with the objective of identifying an area where it was possible to have people speaking Fulfulde and Wolof. The pilot testing was conducted with 50 participants. Pilot testing assessed the feasibility of administering the proposed measures, the administration time and the psychometric properties of the measures. Analysis of the pilot test data did not indicate the need for revisions and/or reductions of the instruments.

### Statistical analysis

Data were managed using Stata version 16 (StataCorp 2019) and analysed using R version 4.1.2. (R Core Team 2021). Respondents’ profile by socio-demographic characteristics and disability status was examined using descriptive statistics. To address the first objective, we started by looking at the distribution of participation among people with and without disability in specific political processes. Because age is a known confounder of the relationship between disability and voting (Afrobarometer 2011:37; Jeroense & Spierings 2023:1–23; Norris 2002:19–34; Tambe & Kopacheva 2024:97–115; World Health Organization 2011, 2015), we used univariate logistic regression models adjusted for age to examine associations between having voted (outcome) and participants’ socio-demographic characteristics as well as their possession of the key documents (birth certificate, national ID card, registration to vote and voters card) and their engagement in specific political processes (interest in discussing politics and membership in a political party). To address the second study objective, we focussed on examining the relationship between disability and having voted, proceeding in two stages. We used a hierarchical regression model-building approach. In the first stage, the relationship was examined, by including all other socio-demographic characteristics in the model (age, sex, education, location, relative wealth). At the second stage, to address the third study objective, we added the possession of key documents, discussing politics and membership of a political party variables. This enabled us to explore if and/or how the relationship between disability and having voted was mediated by types of political engagement. All analyses were conducted using a 5% significance level; no imputation of missing data was done.

### Ethical considerations

This article followed all ethical standards for research on human or animal subjects and have received the ethical approval for this study was obtained from the National Ethics Committees of both countries: Cameroon (reference number: 2021/02/1338/CE/CNERSH/SP) and Senegal (reference number 00000085/MSAS/CNERS/SP of 08 June 2021). Participation in the study was voluntary, and no data were collected before consent was obtained. Confidentiality was maintained at all times. Informed consent from all participants was obtained and documented. All participants were informed in their preferred language (French, English, Fulfulde in Cameroon and French and Wolof in Senegal) about the objectives of the study, the voluntary and confidential nature of the participation, the types of questions asked and the risks and benefits of participating in the study. Specific adaptations and supports were provided to enable people with disabilities to participate fully and safely in this research. For example, a sign language translator was recruited to assist data collectors where participants were deaf. For participants with intellectual difficulties or communication difficulties, a member of the household who understood the participant was invited to assist the data collector during the interview with the participant’s consent (Pente et al. 2022a; 2022b).

## Results

### Participant characteristics

A total of 4180 individuals were included in the analysis in Cameroon. The majority of the participants were from Yaoundé (56.76%), followed by Maroua (34.68%) and Mbalmayo (8.56%). Most participants were aged between 21–39 years (66.21%), age ranged from 21 to 99 years. Most were female (57.58%) and those who completed secondary education were the largest group (43.80%) (Table 1).

**TABLE 1:** Participants characteristics.

Characteristic	Variable	Cameroon		Senegal	
		*n*	%	*n*	%
Age group (years)†	≤ 29	1597	39.89	1397	41.27
	30–39	1131	26.32	927	19.63
	40–49	594	13.82	660	13.97
	50–59	384	8.94	497	10.52
	60–69	314	7.31	408	8.64
	≥ 70	160	3.72	282	5.97
Sex	Male	1769	42.42	1493	36.14
	Female	2411	57.58	2678	63.86
Highest level of education	Never went to school	548	12.87	1650	37.14
	Primary	1018	23.92	1107	26.00
	Secondary	1811	43.80	953	26.47
	University or other‡ or technical college	803	19.41	461	10.40
Relative wealth quintile	Q1–Q2 (poorest)	1324	31.65	706	17.19
	Q3- Q4- Q5 (wealthiest)	2856	68.35	3465	82.81

† , For Cameroon, the first age group start from 21–29 years and for Senegal 20–29 years;

‡ , Only 1 person answered ‘other’ therefore we will refer to this category as ‘university’ in the rest of the paper.

In Senegal, the analyses were conducted on 4171 individuals. The majority of the participants were from Kaolack (50.90%) followed by Louga (21.79%), Pikine (15.29%) and finally Kaffrine (12.03%). Most participants were aged between 20 and 39 years (60.9%), age ranged from 20 to 98 years. The majority were female (63.86%) and those with no formal education constituted the largest group (37.14%) (Table 1).

In Cameroon, study participants were slightly wealthier than the average urban population in Cameroon (31.7% in the two poorest quintiles against the expected 40%, if they were similar to the average urban population of the country). In Senegal, study participants were substantially wealthier than the average urban population (17.2% in the two poorest quintiles against the expected 40%).

### Prevalence of disability

Overall, the sample prevalence of disability was 9.77% in Cameroon and 10.89% in Senegal. The prevalence of disability starkly increased with age in both countries. It was higher among women than men in both the countries: 11.48% versus 7.46% in Cameroon, respectively; and 12.24% versus 8.49% in Senegal, respectively. There were also regional variations in both the countries (Table 2).

**TABLE 2:** Sample prevalence of disability.

Sample	Variable	Cameroon			Senegal		
		*n*	%	95% CI	*n*	%	95% CI
Overall	-	412	9.77	8.30, 11.47	494	10.89	9.39, 12.59
Age group (years)	20–29	72	4.67	3.49, 6.22	68	4.52	3.38, 6.01
	30–39	85	7.52	5.82, 9.65	55	5.93	4.27, 8.19
	40–49	57	9.60	7.35, 12.43	60	9.09	7.03, 11.69
	50–59	60	15.63	11.92, 20.21	89	17.91	14.24, 22.27
	60–69	68	21.66	17.22, 26.86	102	25.00	19.48, 31.47
	≥ 70	70	43.75	35.79, 52.05	120	42.55	36.69, 48.63
Sex	Female	279	11.48	9.56, 13.72	356	12.24	10.23, 14.57
	Male	133	7.46	6.06, 9.16	138	8.49	7.23, 9.96
Location (Cameroon)	Maroua	117	7.92	5.53, 11.22	-	-	-
	Mbalmayo	31	8.42	5.73, 12.23	-	-	-
	Yaoundé	264	11.11	9.15, 13.42	-	-	-
Location (Senegal)	Kaffrine	-	-	-	28	5.46	3.81, 7.76
	Kaolack	-	-	-	215	9.15	7.79, 10.73
	Louga	-	-	-	121	12.16	8.73, 16.68
	Pikine	-	-	-	130	19.11	15.01, 24.02

CI, confidence interval.

### Overall political participation

Overall, more than half of the participants had voted in the most recent elections in both Cameroon (52.31%) and Senegal (58.27%). The vast majority of participants had a birth certificate or ID in both countries: 84.98% and 85.45% in Cameroon; 95.93% and 82.77% in Senegal, respectively.

In Cameroon, only around half of the participants were currently registered to vote (55.81%) or had a valid voter card (51.41%), while in Senegal, around two-thirds were registered to vote (66.3%) or had a valid voting card (69.37%). In Cameroon, less than half of the participants (40.62%) owned four key documents (birth certificate, a national ID card, registration to vote and a valid voter card), while in Senegal, 68.19% of participants had all four documents (Table 3).

**TABLE 3:** Overall political participation.

Variable	Cameroon		Senegal	
	*n*	%	*n*	%
**Recently voted**	**2198**	**52.31**	**2746**	**58.27**
**Key documents†**				
Has a birth certificate	3544	84.98	4024	95.93
Has an identification card	3613	85.45	3747	82.77
Is registered to vote	2379	55.81	3047	66.31
Has a valid voting card	2193	51.41	3211	69.37
Has all four key documents	1729	40.62	3158	68.19
**Political engagement**				
Interest in politics‡	2648	63.14	1837	41.39
**If yes, main source of information**				
Television	1285	48.47	622	33.73
Friends and family	541	20.24	360	19.61
Radio	358	13.30	287	15.30
Internet	384	15.04	225	12.83
Other§	80	2.96	343	18.53
**Political party membership**				
Member of a political party†	861	20.25	518	11.16

† , The following variables are all binary, taking the values Yes or No, we have tabulated here the Yes category;

‡ , Interest in politics relate to: watches or listens or discusses;

§ , Other relate to: Community or religious leaders or organisation of persons with disabilities (OPD) or non-governmental organisation(NGO)/local council or newspapers, etc. This category was created by collapsing categories with a proportion of respondents < 10%.

In Senegal, less than half of the participants reported an interest in discussing politics (41.39%), while this was close to two-thirds in Cameroon (63.14%). Among those interested in politics, television (TV) was most commonly cited source of information in both countries: Cameroon (48.57%) and Senegal (33.73%) (Table 3).

The proportion of participants, who are members of a political party was 20.25% in Cameroon and 11.16% in Senegal (Table 3).

### Political participation by disability status

In Cameroon, the proportion of people who recently voted was slightly higher among those with disabilities (57.61%), compared to those without disabilities (51.75%). A significantly lower proportion of people with disabilities (75.24%) had a birth certificate (versus 86.04% in those without disabilities), but the proportion of those having all four documents was similar among people with and without disabilities. There was a significant difference in terms of registration to a political party: respectively, 27.88% and 19.43% of people with and without disability were members of a political party (Table 4).

**TABLE 4:** Political participation by disability status.

Variable	Cameroon				Senegal			
	With disability		Without disability		With disability		Without disability	
	*n*	%	*n*	%	*n*	%	*n*	%
**Recently voted**	**230**	**57.61**	**1968**	**51.75**	**313**	**60.89**	**2433**	**57.96**
**Key documents**								
Has a birth certificate	310	**75.24**	3234	86.04*	469	94.93	3555	96.05
Has an identification card	343	82.14	3270	85.81	448	89.04	3299	82.01
Is registered to vote	241	58.17	2138	55.56	339	67.12	2708	66.21
Has a valid voting card	229	55.42	1964	50.98	378	74.31	2833	68.78
Has all four key documents	167	40.34	1562	40.65	370	72.60	2788	67.66
**Political engagement**								
Interest in politics	240	59.42	2408	63.54	230	47.04	1607	40.71
Registered member of a political party	115	**27.88**	746	19.43*	69	13.61	449	10.86

Note: Elements in bold with * denote a significant difference between people with and without disabilities (*p* < 0.05).

In Senegal, there was no significant difference between those with and without disabilities in terms of recent voting (60.89% and 57.96%). No other significant difference was observed (Table 4).

### Disability status and voting in recent elections

In both countries, univariate regression models adjusting for age showed that there were significant associations between recent voting and disability status, education, location, interest in politics, possession of all key documents and membership of a political party (*p* < 0.01 for all) (Table 5). Indeed, people with disabilities were less likely to have recently voted than those without disabilities (in Cameroon, OR = 0.52 95% Confidence Interval [CI] = [0.37, 0.73], *p* < 0.01), in Senegal, OR = 0.36, 95% CI = [0.27, 0.48], *p* < 0.01). In Cameroon, despite the significant association between recent voting and highest level of education, no meaningful pattern was observed. However, in Senegal, people with formal education were more likely to have recently voted than those without formal education, with ORs ranging from 1.66 for those who completed primary to 2.35 for those who completed university, *p* < 0.01) (Table 5). People living in Yaoundé or Mbalmayo were less likely to have recently voted than those living in Maroua (OR = 0.53 [0.41, 0.69] and OR = 0.65 [0.44, 0.96], respectively). While in Senegal, people who lived in Kaolack were significantly less likely to have recently voted than those living in Kaffrine (OR = 0.64 [0.48, 0.84]).

**TABLE 5:** Univariate associations with recent voting, results of models adjusted for age.

Covariate	Cameroon			Senegal		
	OR	95% CI	*p*	OR	95% CI	*p*
Female (vs male)	0.53	0.46, 0.61	< 0.01	0.96	0.84, 1.10	0.56
With disability (vs without disability)	0.52	0.37, 0.73	< 0.01	0.36	0.27, 0.48	< 0.01
**Highest level of education (ref: Never went to school)**	**-**	**-**	**0.01**	**-**	**-**	**< 0.01**
Primary	1.28	0.95, 1.71	-	1.66	1.38, 2.00	-
Secondary	0.94	0.70, 1.27	-	1.17	0.94, 1.44	-
University	1.05	0.76, 1.46	-	2.35	1.83, 3.02	-
Relative wealth: Wealthier Q3–Q5 (vs poorest Q1–Q2)	1.00	0.80, 1.24	0.99	-	-	0.45
**Location (ref: Maroua)**	**-**	**-**	**< 0.01**	**-**	**-**	**-**
Mbalmayo	0.65	0.44, 0.96	-	-	-	-
Yaounde	0.53	0.41, 0.69	-	-	-	-
**Location (ref: Kaffrine)**	**-**	**-**	**-**	**-**	**-**	**< 0.01**
Kaolack	-	-	-	0.64	0.48, 0.84	-
Louga	-	-	-	0.86	0.65, 1.14	-
Pikine	-	-	-	0.79	0.55, 1.14	-
Interest in politics: Yes (vs no)	3.67	3.00, 4.49	< 0.01	1.99	1.75, 2.26	< 0.01
Possession of all key documents: Yes (vs. no)	34.41	26.84, 44.13	< 0.01	45.83	36.73, 57.19	< 0.01
Membership of a political party: Yes (vs no)	10.81	7.88, 14.85	< 0.01	2.60	2.13, 3.18	< 0.01

OR, Odds Ratio; CI, Confidence Interval; ref, reference.

Finally, as shown in Table 5, in Cameroon, results show that participants interested in politics, having all the key documents or registered with a political party were more likely to have recently voted (respectively, OR = 3.67 [3.00, 4.49]), OR = 34.41 [26.84, 44.13]) and OR = 10.81 [7.88, 14.85]). Results showed similar associations, although with slightly different order of magnitudes, in Senegal (respectively, OR = 1.99 [1.75, 2.26], OR = 45.83 [36.73, 57.19] and OR = 2.60 [2.13, 3.18]).

After adjusting for socio-demographic characteristics in a multivariable model, the association between disability and having recently voted remained significant and of a similar magnitude as in the univariate analysis for both Cameroon (OR = 0.58 [0.40, 0.84], < 0.01) and Senegal (OR = 0.36 [0.26, 0.44], *p* < 0.01) (Table 6). This suggest that disability status is an important factor associated with participation in elections. In both countries, people with disabilities were less likely to participate in the most recent elections independently of their age, sex, education, location or relative wealth.

**TABLE 6:** Association between disability and recent voting- results of multivariable logistic model including socio-demographic variables.

Covariate	Cameroon			Senegal		
	OR	95% CI	*p*	OR	95% CI	*P*
With disability (vs without disability)	0.58	0.40, 0.84	< 0.01	0.34	0.26, 0.44	< 0.01
Age	1.09	1.07, 1.10	< 0.01	1.06	1.05, 1.07	< 0.01
Female (vs male)	0.56	0.48, 0.65	< 0.01	1.08	0.93, 1.26	0.31
**Highest level of education (ref: Never went to school)**	-	-	0.02	-	-	< 0.01
Primary	1.55	1.17, 2.05	-	1.69	1.42, 2.00	-
Secondary	1.41	1.04, 1.90	-	1.18	0.97, 1.44	-
University	1.55	1.06, 2.26	-	2.36	1.81, 3.09	-
**Relative wealth: Wealthier Q3–Q5 (versus poorest Q1–Q2)**	1.09	0.86, 1.39	0.48	1.01	0.80, 1.29	0.90
**Location (ref: Maroua)**	-	-	< 0.01	-	-	-
Mbalmayo	0.63	0.42, 0.94	-	-	-	-
Yaounde	0.49	0.36, 0.65	-	-	-	-
**Location (ref: Kaffrine)**	-	-	-	-	-	< 0.01
Kaolack	-	-	-	0.65	0.49, 0.85	-
Louga	-	-	-	0.89	0.68, 1.18	-
Pikine	-	-	-	0.84	0.58, 1.22	-

OR, Odds Ratio; CI, Confidence Interval; vs, versus; ref, reference.

When additionally including other political participation variables (interest in politics and membership of a political party), in the multivariable model, the magnitude of association between disability and voting decreased and reached the threshold of statistical significance in Cameroon (OR = 0.64 [0.41, 1.01], *p* = 0.05). In Senegal, the association remained highly significant (OR = 0.38 [0.27, 0.53], *p* < 0.01) (Table 7). Results from this second stage of multivariate analysis indicate that, in both countries, the presence of a severe functional difficulty impacts negatively on one’s ability to participate in elections, independently of their individual characteristics or of their engagement in politics.

**TABLE 7:** Association between disability and recent voting – results of multivariable logistic model including socio-demographic and political engagement variables.

Covariate	Cameroon			Senegal		
	OR	95% CI	*p*	OR	95% CI	*p*
With disability (vs without disability)	0.64	0.41, 1.01	0.05	0.38	0.27, 0.53	< 0.01
Age	1.06	1.05, 1.07	< 0.01	1.05	1.04, 1.06	< 0.01
Female (vs male)	0.86	0.70, 1.06	0.15	1.14	0.96, 1.35	0.14
**Highest level of education (ref: Never went to school)**	-	-	**< 0.01**	-	-	**< 0.01**
Primary	0.91	0.65, 1.28	-	1.22	0.99, 1.51	-
Secondary	0.48	0.33, 0.69	-	0.83	0.63, 1.08	-
University	0.38	0.24, 0.60	-	1.31	0.94, 1.83	-
Relative wealth: wealthier (Q3 – Q5 vs poorest Q1–Q2)	1.09	0.84, 1.41	0.53	0.98	0.71, 1.34	0.88
**Location** (ref: Maroua)	-	-	**< 0.01**	-	-	-
Mbalmayo	0.57	0.42, 0.78	-	-	-	-
Yaounde	0.39	0.30, 0.52	-	-	-	-
**Location (ref: Kaffrine)**	-	-	-	-	-	0.19
Kaolack	-	-	-	0.88	0.61, 1.28	-
Louga	-	-	-	1.11	0.78, 1.60	-
Pikine	-	-	-	1.03	0.64, 1.66	-
Interest in politics: Yes (vs no)	3.09	2.34, 4.07	< 0.01	1.65	1.34, 2.04	< 0.01
Possession of all key documents: Yes (vs no)	37.51	27.76, 50.68	< 0.01	40.41	32.08, 50.90	< 0.01
Registered as member of a political party: Yes (vs no)	6.36	4.23, 9.56	< 0.01	2.42	1.72, 3.40	< 0.01

OR, Odds Ratio; CI, Confidence Interval; vs, versus; ref, reference.

## Discussion

The aim of this study was to assess levels of political participation among people with and without disabilities and explore factors associated with voting in urban populations in Cameroon and Senegal. Guided by Van Deth’s conceptual map of political participation (Van Deth 2014:349–367) and the Civic Voluntarism Model’s determinants of voting behaviour (Barkan 2004:913–937; Verba et al. 1995), we focussed on socio-demographic characteristics and other types of political engagement, such as voter registration, involvement in political discussions and political party membership.

Overall, in both countries, slightly more than half of the participants of eligible age had voted in the most recent elections. In both countries, voting behaviour was strongly associated with older age. The finding is consistent with many other studies. Norris (2002) identified age as one of the most important demographic factors influencing voter turnout and reported that young people generally showed lower interest in voting (Norris 2002:19–34). In Africa, data from the Afrobarometer also showed that youth tend to vote less and express a lower level of partisanship (Afrobarometer 2011:37)

For other individual characteristics, women were less likely to vote only in Cameroon. One possible explanation for this finding could be the patriarchal nature of the Cameroonian society (Nguindip 2023:37–50), which may be related to cultural or religious beliefs that have been shown to hamper efforts aimed at gender equality (Tambe & Jormfeldt 2024: 1–27). Other studies in the region have also shown differences between women and men in the way they vote (Afrobarometer 2021:236). Education was associated with voting in both Senegal and Cameroon when other socio-demographic factors were accounted for, with people, who never went to school being less likely to report voting than people with education. We did not find any association between voting and relative wealth.

Availability of the key documents, such as birth certificates and national ID cards, is often reported as vital for electoral registration and voting (Electoral Institute for the Sustainability of Democracy in Africa 2010) (Ayang Macdonald 2022; Government Accountability Office 2014). For example, in Cameroon, to register on electoral list one has to present their ID card and a voter card will be issued. The Carter Centre study on voter ID published in 2013 highlighted that the main barrier to obtaining a voter card was the difficulties in obtaining the national ID card for those, who did not have birth certificates, as they had to apply to court to obtain a nationality certificate, while a birth certificate was costly to obtain for those, who missed the initial birth registration process of 3 months (Carter Centre 2013). The situation appears to be less challenging in Senegal, where a national biometric identity card with a chip was adopted in 2005 (Passanti 2021:515–525) to act as ID card and voter card. It is the only document accepted to identify the elector (Direction Générale des Elections 2018).

Our results show that in both countries, most participants had the civic documents required (birth certificate, ID card). However, not all those holding the required documents had registered to vote or had a voter’s card. In Senegal, a higher proportion of participants was registered to vote and had a voter card than in Cameroon, most likely because of a better chip-based registration system described earlier (Passanti 2021:515–525). In this setting, there was about 8% gap between those registered to vote and voting in the past election. In the multivariate analysis, the possession of all four documents was associated with higher likelihood of voting in the past election.

We also found a significant association between expressed interest in politics and voting at the last election in both countries. Similar results were reported in other studies, which showed that political interest is one of the main predictors of electoral participation (Goldberg & Sciarini 2023:141–160; Smets & Van Ham 2013:344–359). We also found that one in ten participants in Senegal and one in five in Cameroon were registered as members of a political party and similarly to other settings (Dallaire 2016), these participants were more likely to vote in the election.

One of our primary interests in this study was to examine associations between voting in the most recent elections and disability. Data on political participation of people with disabilities can be difficult to interpret and compare across settings. Firstly, disability is a complex concept, which is defined and measured differently. Secondly, and more importantly, as shown earlier, there are a number of factors associated with political participation and many of them are associated with disability. These include age (disability increases sharply with age and older people tend to be more politically active; Afrobarometer 2011:37; Norris 2002:19–34); sex (disability prevalence is higher among women, and women tend to be less interested in politics than men); education (people with disabilities have lower levels of education and people with lower levels of education are less politically active) and wealth (people with disabilities have lower socio-economic status and people with lower socio-economic status show lower levels of political engagement). It is therefore critical to always specify how disability status was established in a study and to adjust study results for age and other relevant confounders.

In this study, we used the Washington Group Questions on Disability, a validated international tool developed to provide comparable disability data. The estimated prevalence of disability in the studied age group was around 10% in both countries, which is consistent with results of other studies using the Washington Group Questions in these settings (Mactaggart et al. 2016:e0164470, 2021:9213). In both countries, as expected, the prevalence of disability was slightly higher among women and increased considerably with age. Other studies showed similar trends (Hughes 2018:644–645; Pili et al. 2018:7; Ritz & Asamoah 2021).

After adjustment for confounding factors, our findings show that people with disabilities were significantly less likely to vote in the most recent elections than people without disabilities in both countries. This suggests that in these settings, the presence of a severe functional difficulty creates a barrier to political inclusion irrespective of other individual characteristics, location, wealth or interest in politics. Lower levels of participation of people with disabilities in elections has been documented previously but largely in high income settings. For example, a review of election data from several states in the United States published in 2021 reported that having a disability decreased voter turnout by 6.4% to 8.9% (Stum 2021:19). In Europe, the analysis of the European Social Survey and the European Union Fundamental Rights Agency data reported the average voting gap of 8.38% in 2016 (Teglbjærg et al. 2022:1342–1361). Lower levels of political participation of people with disabilities are often attributed to environmental factors, such as lack of accessible communication, unadjusted polling stations and negative social attitudes. The impact of such factors in resource constrained settings of low- and middle-income countries (LMICs) is likely to be more severe than in high-income countries.

Overall, the results of our study support the utility of the civic voluntarism model (Barkan 2004:913–937; Verba et al. 1995) and highlight specific variables and components that help explain voting participation of the studied populations. We show that age, possession of key documents and interest in politics impact voting behaviour, and disability status has a significand effect independent of other factors.

Equal participation in decision-making is the cornerstone of any democratic society, as the collective and active commitment of each citizen fosters collective accountability and social transformation (Krishna 2002:437–460). Therefore, addressing the factors that have been highlighted here as associated with voting (possession of key documents, interest in politics, etc.) is likely to enhance the overall levels of voting. However, addressing environmental factor such as registration and polling station accessibility, social attitudes and access to political information is critical to close disability gaps in voting and ensure equitable opportunities and levels of political participation between people with and without disabilities. Further research on the pathways for disability exclusion and how they affect people with different types of functional difficulties is also needed.

Our study has some limitations. Firstly, this was a cross-sectional study that is limited in establishing temporal relationship between the covariates and the primary outcome. Secondly, the study was conducted in specific urban areas that were also the implementation sites for political participation programmatic activities led by Sightsavers, therefore the findings cannot be generalised. Thirdly, the study was conducted during coronavirus disease 2019 (COVID-19) and as a result of the preventive measures in place at the time, we faced several hurdles in data collection and some participants were not able to participate. This might have affected the quality of the data collected and the generalisation of the results.
