# Corrigendum: Electoral participation of people with and without disabilities in urban communities in Cameroon and Senegal

**DOI:** 10.4102/ajod.v14i0.1714

**Published:** 2025-04-17

**Authors:** Vladimir Y. Pente, Anita Jeyam, Stevens Bechange, Emma Jolley, Anne Roca, Sandra R. Dossou, Khady Ba, Joseph Oye, Salimata Bocoum, Laurene Leclercq, Elena Schmidt

**Affiliations:** 1 Sightsavers, Cameroon Country Office, Yaoundé, Cameroon; 2 Sightsavers, Haywards Heath, United Kingdom; 3 Sightsavers, Senegal Country Office, Dakar, Senegal

In the published article, Pente, V.Y., Jeyam, A., Bechange, S., Jolley, E., Roca, A., Dossou, S.R. et al., 2024, ‘Electoral participation of people with and without disabilities in urban communities in Cameroon and Senegal’, *African Journal of Disability* 13(0), a1399. https://doi.org/10.4102/ajod.v13i0.1399, the ORCID of Laurene Leclercq was given incorrectly in the ‘Authors’ section.

Instead of https://orcid.org/0000-0001-9466-0883, the correct ORCID for author, Laurene Leclercq, should be: https://orcid.org/0009-0008-4956-0179.

The authors apologise for this error. The correction does not change the study’s findings, its significance or overall interpretation of the study’s results or the scientific conclusions of the article in any way.

